# 465. Use of Monoclonal Antibodies for COVID-19 in Solid Organ Transplant Recipients: A Single-Center Experience

**DOI:** 10.1093/ofid/ofad500.535

**Published:** 2023-11-27

**Authors:** Keven Gomez, Jonathan T Arcobello, Sandy Chang

**Affiliations:** University of California San Diego Health, San Diego, California; Loma Linda University Medical Center, Loma Linda, California; Loma Linda University, Loma Linda, California

## Abstract

**Background:**

The use of coronavirus disease 2019 (COVID-19) monoclonal antibody (mAb) treatment in solid organ transplant (SOT) patients have been shown to reduce the rates of hospitalization, Emergency Department (ED) visits, and mortality in previous studies. However, most of these studies provide little or no data in minority patients, specifically Hispanic patients, a group that has been associated with worse outcomes with COVID-19. Our study describes the use of COVID-19 mAbs in the SOT recipients at our institution, where a majority of patients are of Hispanic ethnicity.

**Methods:**

We performed a retrospective chart review of all adult SOT patients who received COVID-19 mAbs from December 2020 to December 2022.

**Results:**

We identified 54 patients who were SOT recipients. The mean age was 46.3 (IQR 33-59), and 30 (55.6%) were male. Of the 54 patients, 34 (63.0%) identified as Hispanic, 17 (31.5%) as White, 2 (3.7%) as Asian, and 1 (1.9%) as Black. There were 23 (42.6%) heart, 22 kidney (40.7%), 6 liver (11.1%), 2 (3.7%) combined kidney and liver, and 1 (1.9%) combined kidney and pancreas recipients. There were four mAbs available during the study period. Of the 54 SOT recipients, 16 (29.6%) received casirivimab/imdevimab, 5 (9.3%) received bamlanivimab, 19 (35.2%) received bebtelovimab, and 14 (25.9%) received sotrovimab. Average time from symptom onset to receipt of infusion was 4 (IQR 1-5) days. The 30-day hospitalization rate due to COVID-19 from time of diagnosis in our cohort was 5.6%. There were 3 (5.6%) patients who visited the ED due to COVID-19 within 30 days of diagnosis and there were no deaths due to COVID-19 within 30 days of diagnosis. When comparing between the Hispanic and non-Hispanic groups in our cohort, the 30-day hospitalization rate due to COVID-19 from time of diagnosis was not statistically significant (5.6% vs 0, p=0.29). There was one patient who had an infusion reaction and 3 patients who developed allograft rejection within 90 days of mAb infusion.

**Conclusion:**

In our small cohort of SOT recipients, the majority of which are Hispanic, use of COVID-19 mAbs had low rate of poor outcomes and adverse events. There was no statistically significant difference in hospitalization rate following mAb infusions between the Hispanic and non-Hispanic SOT recipients in our group.

**Disclosures:**

**All Authors**: No reported disclosures

